# Cisplatin induces hippocampal neurotoxicity and cognitive impairment in rats through neuroinflammation, oxidative stress, and overexpression of glutamatergic receptors mRNA

**DOI:** 10.3389/fphar.2025.1592511

**Published:** 2025-05-30

**Authors:** Ahmad Hamad Alhowail

**Affiliations:** Department of Pharmacology and Toxicology, College of Pharmacy, Qassim University, Buraydah, Saudi Arabia

**Keywords:** cisplatin, hippocampus, inflammation, glutamate receptors, oxidative stress

## Abstract

Chemotherapy-induced cognitive deficits are a prevalent adverse effect in patients with cancer undergoing chemotherapy. We investigated cisplatin-induced neurotoxicity by assessing neuroinflammation and expression of glutamate receptors. Two groups of eight-week-old rats (n = 10 per group) were used: control and cisplatin-treated. Cisplatin (8 mg/kg, i. p.) was administered each 2 days for three cycles. From rats hippocampi, we measured: concentrations of nuclear factor-kappa B (NF-κB), tumor necrosis factor (TNF)-α, and interleukin (IL)-6); mRNA countenance of synapse-related proteins (α-Amino-3-hydroxy-5-methyl-4-isoxazolepropionic acid receptors (AMPARs) and N-methyl-D-aspartic acid receptors (NMDARs); levels of reactive oxygen species (ROS), nuclear factor erythroid 2-related factor 2 (Nrf-2), superoxide dismutase (SOD); mitochondrial complex I (MCI) activity; lipid peroxidation. The cisplatin group exhibited significant reductions in survival rate to 40% and body weight, confirming the initiation of cisplatin toxicity. In contrast with the control group, the cisplatin group exhibited notably increased hippocampal levels of pro-inflammatory substances (NF-κB, TNF-α, IL-6), synapse-related proteins (AMPARs, NMDARs), and oxidative-stress mediators (ROS, Nrf-2, SOD). Cisplatin treatment resulted in declined MCI activity and increased lipid peroxidation. These findings indicate that cisplatin-induced cognitive impairment may be mediated by heightened hippocampal neuroinflammation and overactivation of glutamatergic receptors.

## Graphical Abstract

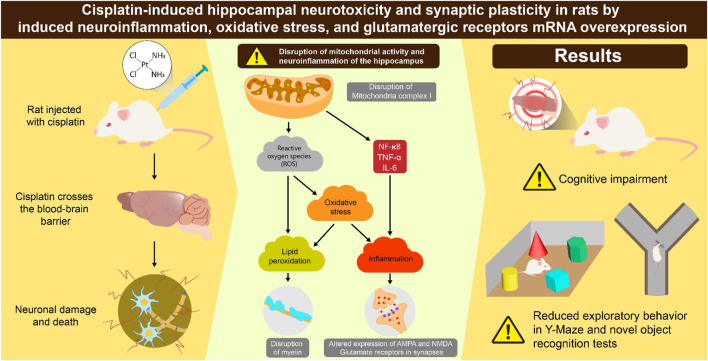

## 1 Introduction

Cisplatin is a frequently utilized medication for cancer therapy (Dasari and Bernard Tchounwou, 2014). The mechanism of action of cisplatin involves an alteration in Ca^2+^ balance, the induction of lipid peroxidation, and the infliction of DNA damage. These actions contribute to the apoptosis of cancer cells (Dasari and Bernard Tchounwou, 2014; Elmorsy et al., 2024). Cisplatin is one of the most efficacious antineoplastic agents (Prieložná et al., 2024). Nonetheless, its extended use is restricted due to the potential for various acute and long-term toxic effects.

The toxicity of cisplatin manifests as cardiovascular and metabolic dysfunction, hypogonadism, peripheral neuropathy, and cognitive deficits (Park et al., 2024). Usually, cisplatin neurotoxicity presents as cognitive impairment (termed “chemo-brain”) even in patients given for tumors apart that the central nervous system (CNS) (Was et al., 2022). Approximately 60%–75% of patients report short/long-term cognitive deficits during cisplatin therapy (Alhowail et al., 2019), and some patients experience nervousness and depression (Országhová et al., 2021).

The processes causing the cognitive deficiency caused by cisplatin are incompletely understood. However, increasing evidence indicates that it resembles “advanced aging of the brain” (Alotayk et al., 2023; Umfress et al., 2021). Furthermore, cisplatin appears to induce structural and functional modifications in the hippocampus, leading to neuropathologic changes (Umfress et al., 2021). The hippocampus is crucial for cognitive functions, encompassing attention, memory, and the processes involved in learning (Rubin et al., 2014). Nonetheless, the hippocampus exhibits considerable vulnerability and sensitivity to anti-cancer agents such as cisplatin, with dysfunction being closely associated with various pathologic conditions (Park et al., 2024; Alotayk et al., 2023). Research indicates that hippocampal dysfunction correlates with a higher risk of mild cognitive deficits and neurodegenerative conditions such as dementia (Hanseeuw et al., 2016).

According to clinical and animal research, systemic inflammation is a significant indicator for the onset of various diseases (Zotova et al., 2023). Increased inflammation is a crucial factor in the advancement of neurodegenerative illnesses, and this has been recognized across various pathways (Adamu et al., 2024). Numerous investigations have implied that cisplatin enhances the diffusion of inflammatory substances, together with tumor necrosis factor-alpha (TNF-α) and interleukins (ILs) (Jang et al., 2021). These mediators play important parts in oxidative injury, mitochondrial impairment, neuronal apoptosis, and changes in neurotransmitter levels within the hippocampus (Wen et al., 2025). Moreover, it has been suggested that synaptic dysfunction (including synapse loss and deficits) is closely linked to cognitive impairment (Lepeta et al., 2016).

The mechanism of action of cisplatin involves damaging DNA, thereby resulting in the accumulation of cellular debris within the cellular matrix. This accumulation heightens inflammation, ultimately resulting in oxidative stress (OS). Typically, increased OS is indicated by increased levels of reactive oxygen species (ROS) and enzymatic antioxidants such as superoxide dismutase (SOD) and glutathione peroxidase (GPX). These enzymes are generated among the creation of nuclear factor erythroid 2-related factor 2 (Nrf-2), a nuclear transcription factor. Nrf-2 interacts with the antisense response element (ARE) promoter region, thus augmenting the expression of SOD and GPX. Reports have indicated that an increase in the ROS level and a reduction in the degrees of SOD and GPX serve as OS markers. Treatment with doxorubicin or cisplatin has been shown to increase the ROS level while diminishing the concentrations of SOD and GPX. Furthermore, cisplatin activates the mitochondrial apoptotic pathway by modulating the expression of apoptotic genes such as p53, Bcl-2, Bcl-2-associated X protein (Bax), and caspases (Li et al., 2024). Additionally, one study indicated that a sudden increase in the level of inflammatory mediators correlated with an increase in expression of the targets for α-Amino-3-hydroxy-5-methyl-4-isoxazolepropionic acid (AMPA) and N-methyl-D-aspartic acid (NMDA) (Thomas et al., 2017; Alhowail and Aldubayan, 2023). This increase in glutamate receptors caused instability in neuronal firing and excessive excitation of neurons, increased Ca^2+^ permeability triggering mitochondrial dysfunction, which ultimately resulted in apoptosis (Alhowail and Aldubayan, 2023; Mattson, 2008). In this context, the heightened inflammatory response induced by cisplatin had a role in alteration of the targets for AMPA and NMDA (i.e., AMPAR and NMDAR, respectively), which are crucial for synaptic plasticity and cognitive functions.

Interestingly, cisplatin has been shown to readily penetrate the blood-brain barrier and cause oxidative stress, mitochondrial dysfunction, and neuroinflammation, resulting in neuronal damage in several brain areas, including the hippocampus and prefrontal cortex, which are critical for cognitive function (Hagiwara et al., 2023; Oliveros et al., 2023). Augmented inflammation and OS are characteristics of cancer development and cognitive impairment (Williams et al., 2018). The inflammatory response following cisplatin treatment involves the modulation of specific cytokines, including nuclear factor-kappa B (NF-κB), TNF-α, and IL-6, additionally to ROS, Nrf-2, and SOD. These actions may increase the manifestation of glutamate receptors, such as AMPARs and NMDARs, which are associated with neuronal toxicity and cognitive impairment (Elmorsy et al., 2024; Yu et al., 2020; Guo et al., 2024). It has been shown that cisplatin alters synaptic plasticity and inhibits neurogenesis, mechanisms intricately involved in memory and learning functions (Dietrich et al., 2006; Lomeli et al., 2017). Several efforts have been undertaken to alleviate cisplatin-induced neurotoxicity and memory impairment; one study suggested that ginsenoside Rb1 restored these effects, possibly by restoring neuronal loss and lowering oxidative stress and neuroinflammation (Chen et al., 2019). Despite the crucial role of AMPA and NMDA receptors in synaptic plasticity and memory formation, there is a limitation of studies investigating the implication of AMPA and NMDA receptor expression in the hippocampus in mediating cisplatin-induced cognitive dysfunction. However, it has been shown that cisplatin treatment produced marked increase in AMPA and NMDA glutamate receptor gene expression though in central nucleus of amygdala (Alhadeff et al., 2015). It is quite interesting to investigate their expression that reveal how cisplatin disrupts glutamatergic signaling, offering insight into potential therapeutic targets to prevent or reverse cognitive decline. Additionally, understanding the molecular processes that underlie cisplatin-induced neurotoxicity and cognitive impairment in rodents is vital because it offers insights into the neuropathological effects of a commonly used chemotherapeutic agent and leads to the advancement of neuroprotective interventions. Thus, investigating these cellular processes in animal models not only strengthens scientific knowledge of chemotherapy-related cognitive impairment (commonly known as “chemo brain”) but also paves the way to discover therapeutic targets to mitigate these side effects, thereby improving the quality of life in cancer patients. Therefore, recent research has focused on the pathophysiologic mechanism underlying cisplatin-induced cognitive impairment (Park et al., 2024; Alotayk et al., 2023), but the precise mechanism is not known.

We assessed how cisplatin contributes to cognitive impairment. We focused on hippocampal damage linked to increased neuroinflammation and increased expression of glutamatergic receptors.

## 2 Materials and methods

**FIGURE 1:**
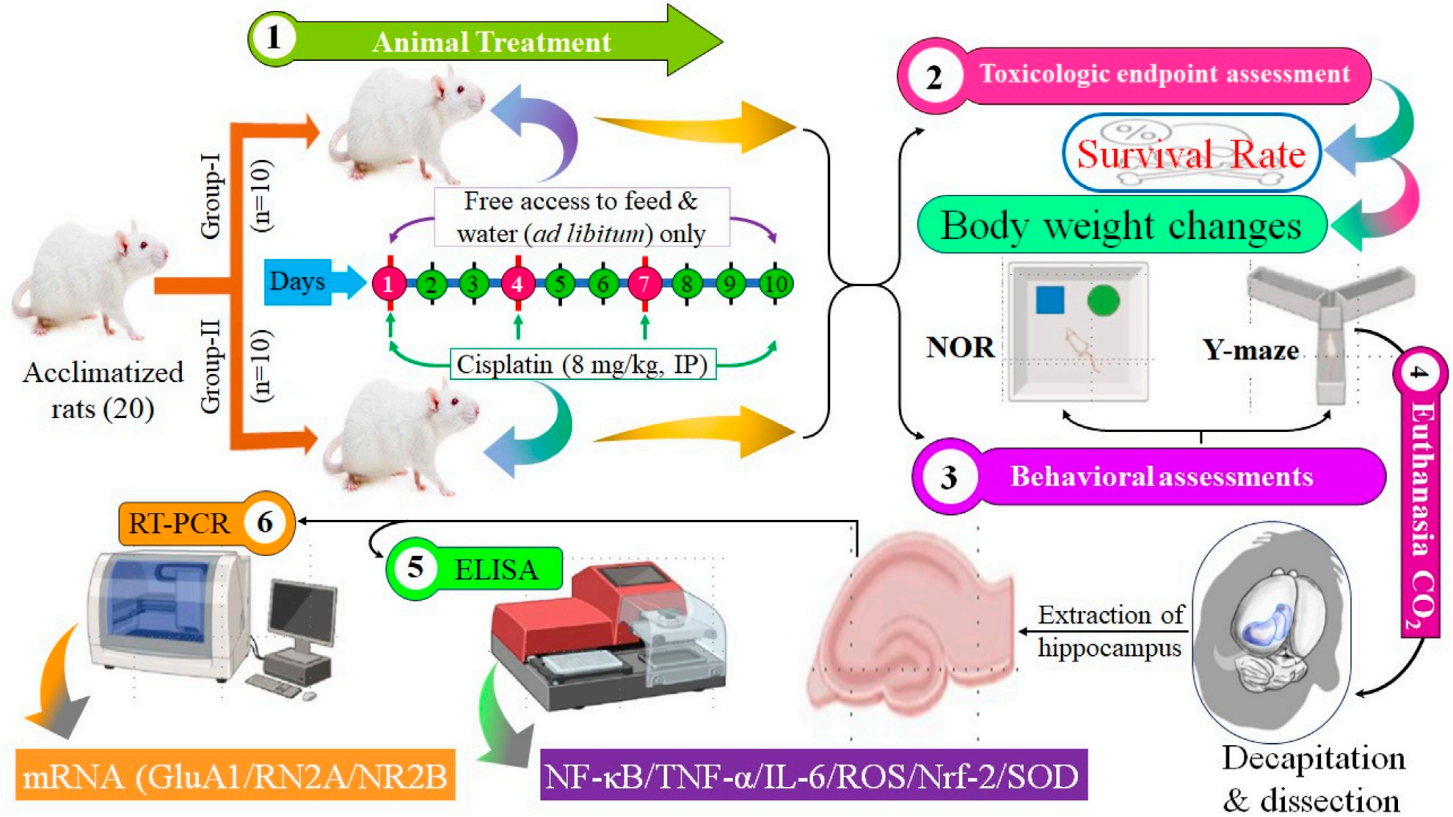
Study design and outcomes (schematic).

The whole study layout is shown in Figure 1.

### 2.1 Drugs and chemicals

Cisplatin (1 mg/mL) was obtained from EBEWE Pharma (Vienna, Austria).

### 2.2 Experimental animals

The Animal Care and Use Committee of Qassim University (Buraydah, Saudi Arabia) permitted the study procedure. Male Wistar rats were acquired from the Animal House of the College of Pharmacy within Qassim University. Twenty rats were allotted randomly to a control group (n = 10) and cisplatin administration group (n = 10). Rats were allocated and housed in four separate cages. Each cage contained five rats to fit the capacity of the cage. Two cages were assigned randomly to the control group and two to the cisplatin-treated group. Food and water were provided daily to ensure adequate hydration and nutrition, promoting responsible animal care throughout the study. All cages were sanitized every 48 h to ensure optimal living conditions. Rats lived in a controlled setting with a 12-h day-night rotation and a temperature of 22°C ± 1.5°C. The protocol was approved by animal ethical committee from the Deanship for Graduate Studies and Scientific Research at Qassim University under number (23-67-05).

### 2.3 Administration of cisplatin and experimental methodologies

Rats received cisplatin (8 mg/kg bodyweight, i. p.) as an administration every 2 days for three cycles (day 1, 4, and 7) to generate long-term hippocampal damage (Abdel-Wahab and Moussa, 2019). The behavioral evaluation was performed on days 8 and 9, while the ELISA and RT-PCR analyses were performed following the euthanasia of the animals on day 11. The comprehensive experimental methodology is illustrated in Figure 1.

### 2.4 Survival rate and body weight

The survival rate was observed daily, providing crucial insights into the ongoing study. Dead animals were removed immediately. Regular monitoring of body weight provides valuable insights into overall health trends. Consistent measurements, taken at 3-day intervals, allow for the identification of subtle changes and help detect potential health issues.

### 2.5 Y-maze test

The Y-maze test (YMT) comprised of three wooden arms measuring 50 cm, 10 cm, and 17 cm in length, every set at a 120° direction from the others. In this experiment, the maze’s innovative arm was deliberately blocked to assess the rats’ memory skills. Each arm was allocated as either the “start,” “familiar,” or “novel” arm. During the exercise phase, a rat was stationed in the starting arm and permitted to explore only the accustomed arm for 10 min. Following a 3-h interim, the test session initiated, during which all arms were reachable. The rat was once again positioned in the starting arm and observed for 3 min to verify whether it showed a preference for the recognizable or novel arm. Lighting specifications in the maze were kept uniform throughout the testing. To measure exploration behavior, video recordings were probed to determine how much time the rats completed in the novel arm and how frequently they entered it. An arm entry was calculated only if all four of the rat’s paws intersected into the arm (Alsaud et al., 2023).

### 2.6 Novel object recognition test

A wooden box determining 40 cm on each side was used to perform the test, including two objects: a acquainted set of black cans and a novel white teacup. During the drill phase, each rat was rested at the center of the box and given 10 min to investigate the familiar black cans. After a 3-h wait, the test phase began, through which the unfamiliar object (teacup) was introduced. The rat was then allowed 3 min to interact freely with both objects. Throughout this period, video recordings were used to assess the time each rat spent investigating the new thing. The resulting data were examined using statistical methods (Alsaud et al., 2023).

### 2.7 Real-time reverse transcription-quantitative polymerase chain reaction

Total RNA was obtained from the brain tissues of rats in both the cisplatin-treated and control groups using TRIzol^®^ Reagent (MilliporeSigma, Burlington, MA, United States). To purge any lingering genomic DNA, the isolated RNA was given with RNase-free DNase (Ambion, Carlsbad, CA, United States). RNA purity was gauged utilizing a NanoDrop ND-2000c spectrophotometer (Thermo Scientific, Waltham, MA, United States). Gene-specific primers (Table 1) were intended across Integrated DNA Technologies (Coralville, IA, United States) and used at a working strength of 10 µM for real-time quantitative PCR (RT-qPCR). Amplification was stocked out using the One-Step SYBR Green RT-qPCR Kit (RK20404; ABclonal Technology, China). Following the manufacturer’s practice, 400 ng of RNA from each trial was reverse transcribed into cDNA and intensified using the AiraMx Real-Time PCR system (Agilent Technologies, Santa Clara, CA, United States). The 20 μL PCR reaction mix encompassed SYBR Green RT-qPCR buffer, ABScript 2 Enzyme Mix, 10 μM forward and reverse primers, ROX II Reference Dye (×50), total RNA, and RNase-free water. The thermal cycling conditions involved one reverse transcription step at 42°C for 5 min, an initial denaturation at 95°C for 1 min, monitored by 40 amplification cycles at 95°C for 5 s and 60°C for 32–34 s. All responses were completed in duplicate throughout three separate experiments. Data were analysed utilizing the AiraMx software (Agilent Technologies) for relative quantification. Gene expression levels were regulated to the housekeeping gene glyceraldehyde-3-phosphate dehydrogenase (GAPDH), and mRNA representation changes were estimated based on relative transcript abundance compared to GAPDH.

**TABLE 1:** Primers employed in our study.

Gene	Sequence (5′–3′)	Length (bp)
Forward: *AMPA-GluA1*	GCC​AGA​TCG​TGA​AGC​TAG​AAA	80
Reverse: *AMPA-GluA1*	CTC​CGC​TCT​CCT​TGA​ACT​TAT​T	
Forward: *NMDA-NR2A*	GGA​GGA​GGT​TGG​GTC​ATT​TAT	86
Reverse: *NMDA-NR2A*	AGT​AGG​CAC​TTG​GGA​CTT​TAC	
Forward: *NMDA-NR2B*	GAG​GAA​CCA​GGC​TAC​ATC​AAA	83
Reverse: *NMDA-NR2B*	GGT​CAC​CAG​GTA​AAG​GTC​ATA​G	
Forward: *GAPDH*	ACT​CCC​ATT​CTT​CCA​CCT​TTG	104
Reverse: *GAPDH*	CCC​TGT​TGC​TGT​AGC​CAT​ATT	

### 2.8 Tissue preparation and enzyme-linked immunosorbent assays

Rats were killed using CO_2_ and decapitated. The brain was washed with frozen oxygenated phosphate-buffered buffer and the hippocampus dissected. Hippocampal tissues were preserved at −80°C until analyses. Before experimentation, hippocampi were homogenized using Neuronal Lysing Buffer with a homogenizer (Q-Sonica, Newtown, CT, United States) and samples centrifuged (13,000 × g, 12 min, 4°C). To gauge the protein density, the supernatant was accumulated using a bicinchoninic acid assay kit (Pierce, Waltham, MA, United States). The quantities of NF-κB, TNF-α, IL-6, ROS, Nrf-2, SOD, mitochondrial complex I (MCI) activity, and lipid peroxidation were assessed in samples utilizing ELISA kits (MyBioSource, San Diego, CA, United States) in agreement with manufacturer guidelines.

### 2.9 Statistical analyses

Statistics are the mean ± standard error of the mean. Graphs were created using Prism 10.4 (GraphPad, La Jolla, CA, United States). Comparison of data involving groups was done using unpaired *t*-tests. *p* ≤ 0.05 was deemed significant.

## 3 Results

### 3.1 Effect of cisplatin on survival

**FIGURE 2:**
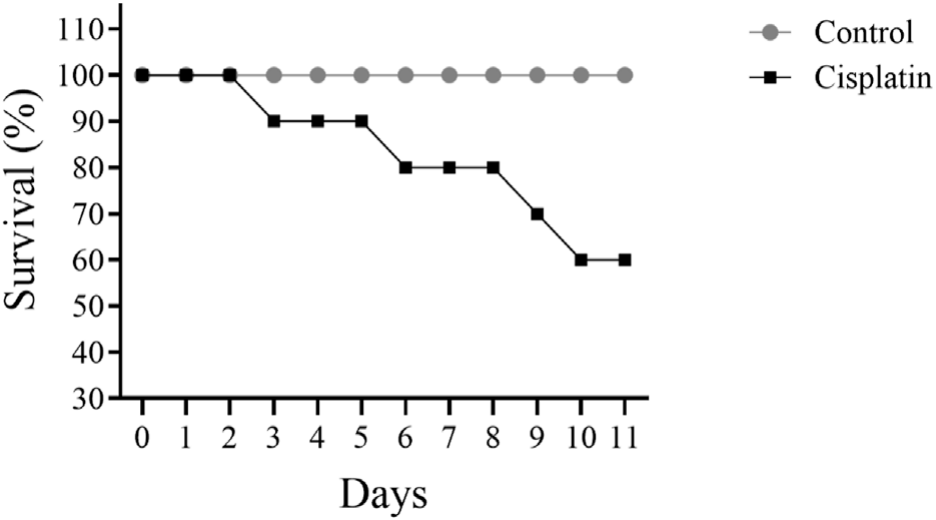
Effect of cisplatin treatment on the survival of rats (n = 10).

Figure 2 illustrates a decline in the figure of rats surviving when comparing cisplatin use to the control grouping. Cisplatin administration led to 40% of rats dying by day 11.

### 3.2 Effect of cisplatin on bodyweight

**FIGURE 3:**
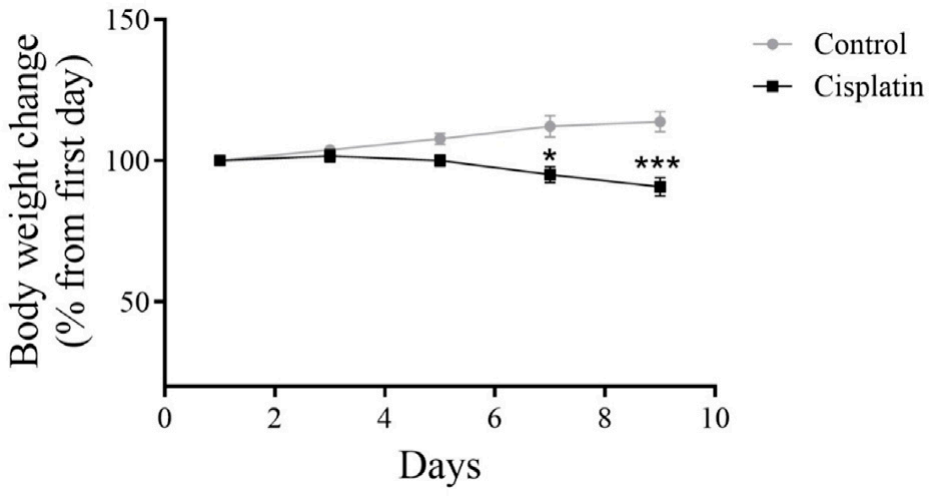
Effect of cisplatin on the bodyweight of rats. We quantified changes in bodyweight from baseline to study completion (n = 6). *p < 0.05 and ***p < 0.001.

To assess the impact of cisplatin toxicity, bodyweight was recorded for rats in the drug induced group and untreated group (Figure 3). In comparison with the control group, a notable drop in bodyweight was observed in the cisplatin group on days 7 and 9 (*p = 0.0104 and ***p < 0.0002, respectively).

### 3.3 Effect of cisplatin on YMT and NORT tasks

**FIGURE 4:**
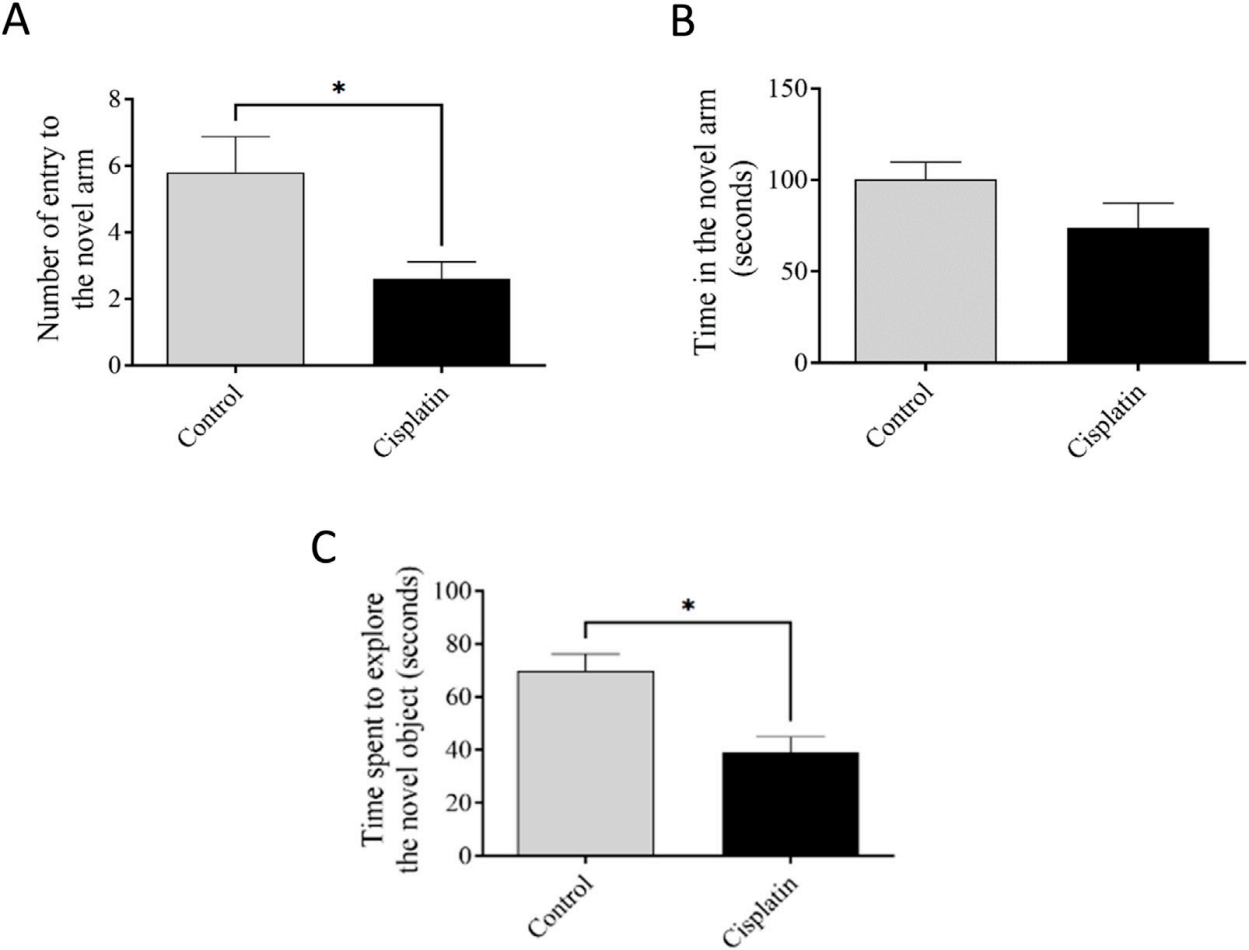
Effect of cisplatin on remembrance of rats. **(A, B)** The sum of accesses and interval occupied in the new arm of the YMT. **(C)** Duration of exploration to a new object in the NORT. *p < 0.05 matched with the untreated group (n = 6).

Figure 4 reveals the performance of rats in the YMT and NORT. There was a decline in the digit of entrances into the narrative arm after induction with cisplatin, but the time interval in the unfamiliar arm was not significant (Figures 4A,B) (*p = 0.0269, and *p = 0.172). The NORT confirmed memory impairment by reducing the exploration time for the novel arm relative to the control (Figure 4C) (*p = 0.0125).

### 3.4 Effect of cisplatin on levels of NF-κB, TNF-α, and IL-6 in hippocampal tissue

**FIGURE 5:**
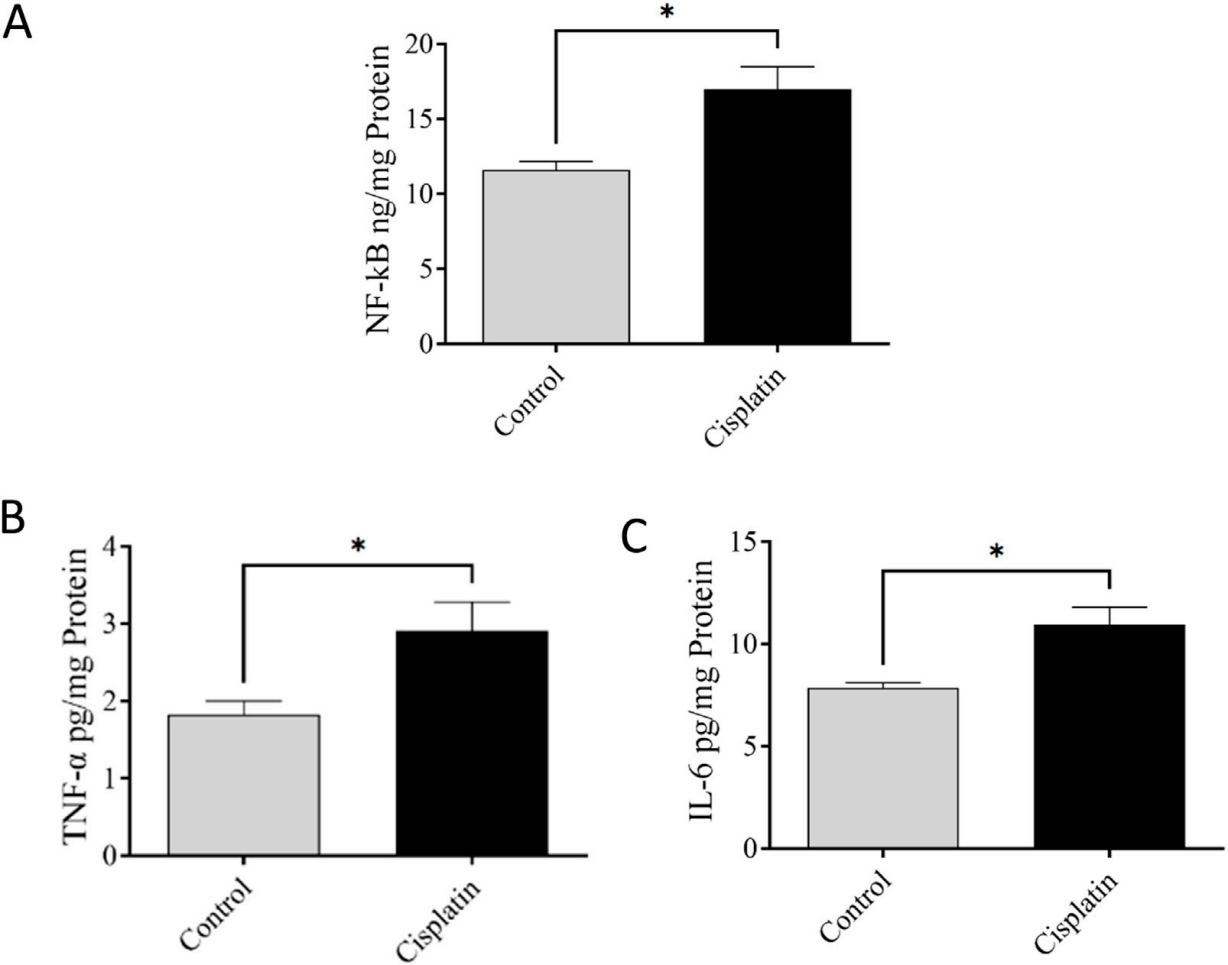
Effect of cisplatin on the hippocampal levels of NF-κB **(A)**, TNF-α **(B)**, and IL-6 **(C)** of rats (n = 6). *p < 0.05.

ELISAs were conducted to quantify amounts of NF-κB, IL-6, and TNF-α. Figures 5A–C demonstrate significantly increased concentrations of NF-κB, TNF-α, and IL-6 (*p = 0.0295, *p = 0.0387, and *p = 0.0137, respectively), in the cisplatin animals in contrast with those in the control rats.

### 3.5 Effect of cisplatin on expression of GluA1, NR2A, and NR2B mRNA in hippocampal tissue

**FIGURE 6:**
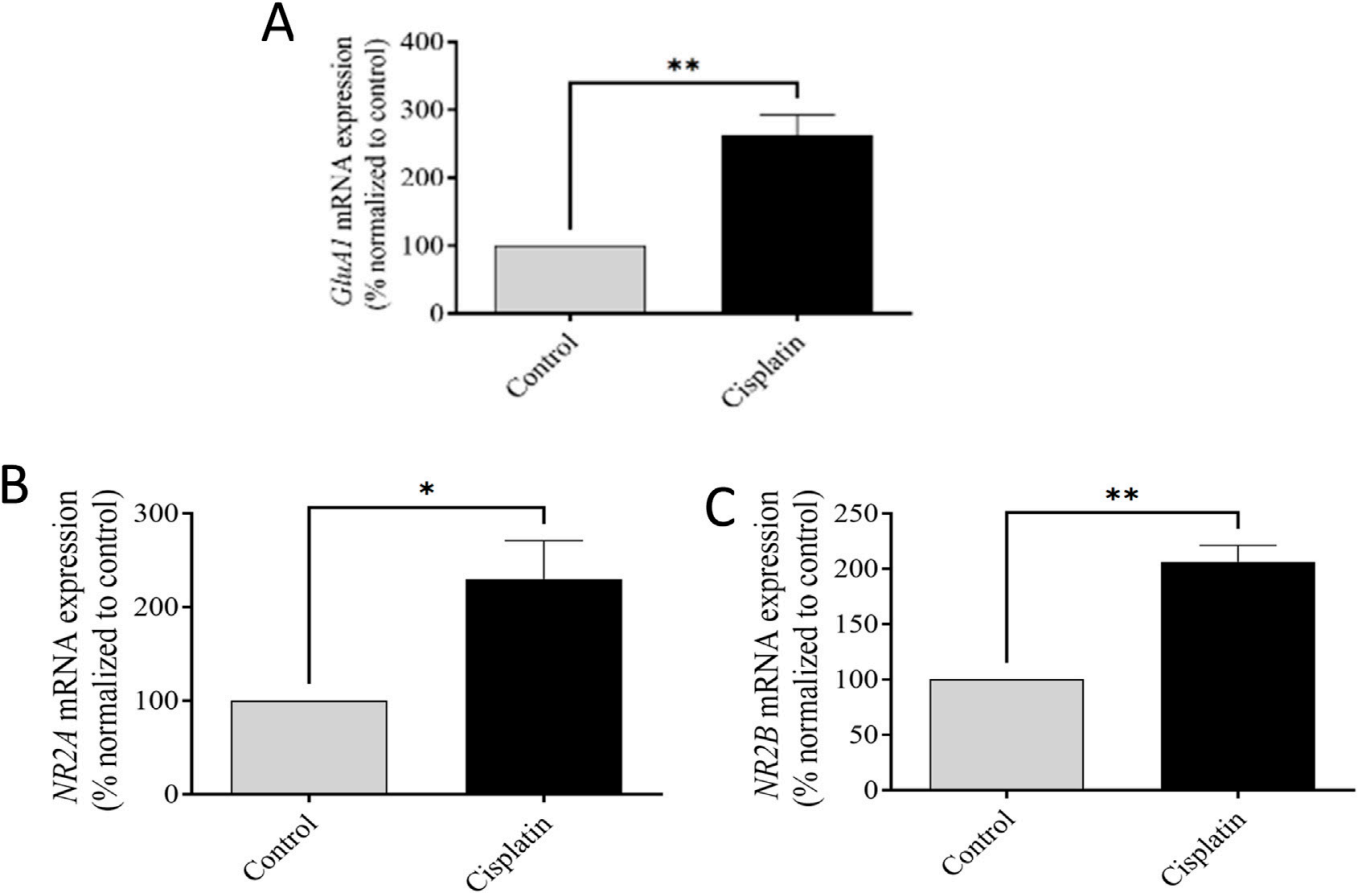
Effect of cisplatin on mRNA representation of the *GluA*1 subunit of AMPAR **(A)** and *NR2A* and *NR2B* subunits of NMDAR in the rat hippocampus **(B, C)**. *p < 0.05 and **p < 0.01 matched with the control (n = 6). All protein data were normalized to the GAPDH and expressed as a percentage of the control group, which was set to 100%.

A difference of mRNA expression of the AMPAR-*GluA1* subunit and NMDAR *NR2A* and *NR2B* subunits were conducted between control and cisplatin-treated hippocampal tissue (Figure 6). Cisplatin treatment resulted in significantly increased mRNA expression of AMPAR-GluA1 (**p = 0.0053) and NMDAR-*NR2A* and -*NR2B* subunits in contrast with those in the control group (*p = 0.0333, and **p = 0.0020 respectively). These discoveries suggested that overexpression of AMPAR-*GluA1* and NMDAR-*NR2A/NR2B* subunit mRNA was associated with cisplatin-induced hippocampal toxicity in rats.

### 3.6 Effect of cisplatin on levels of ROS, Nrf-2, and SOD in hippocampal tissue

**FIGURE 7:**
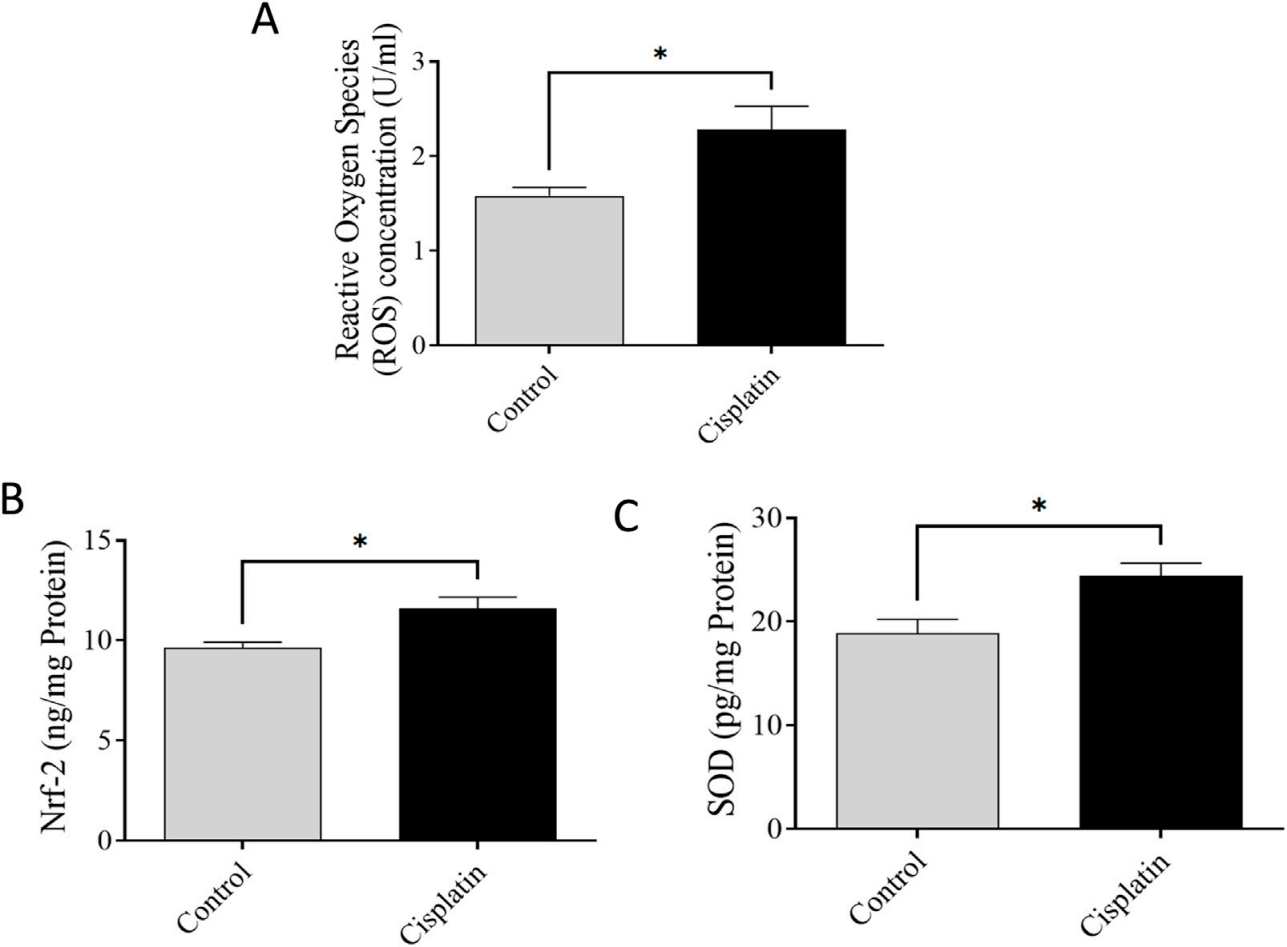
Effect of cisplatin on hippocampal levels of ROS **(A)**, Nrf-2 **(B)**, and SOD **(C)** in rats (n = 6) *p < 0.05.

ELISAs were conducted to quantify levels of ROS, Nrf-2, and SOD. Figures 7A–C demonstrate significantly increased concentrations of ROS, Nrf-2, and SOD, (*p = 0.0369, *p = 0.0375, and *p = 0.0228, respectively), in the cisplatin group in contrast with the normal rats.

### 3.7 Effect of cisplatin on MCI activity and lipid peroxidation in hippocampal tissue

**FIGURE 8:**
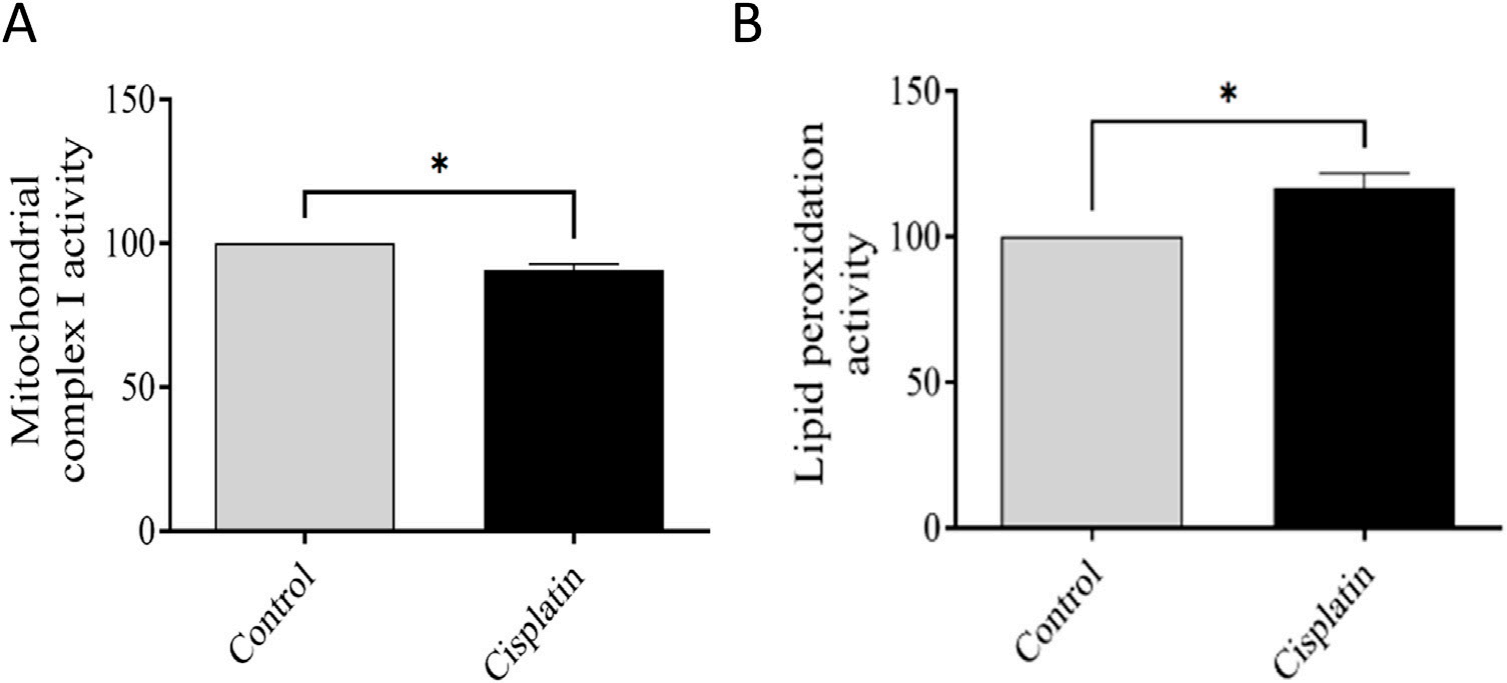
Effect of cisplatin on the action of mitochondrial complex I (MCI) and lipid peroxidation. **(A)** Percentage change in MCI activity relative to the control. **(B)** Percentage change in lipid peroxidation matched to the normal condition (n = 6). *p < 0.05. All protein data were normalized to the controls and expressed as a percentage of the control group, which was set to 100%.

We wished to elucidate the role of mitochondria. Evaluation of MCI activity was conducted, and indicated a deficiency in the oxidized form of NADH. Hence, mitochondrial activity had been disrupted (*p = 0.0130). To validate this impairment (which typically leads to increased free radicals), lipid peroxidation was evaluated. We documented increased lipid peroxidation in hippocampal tissues from cisplatin-treated rats in contrast with that in the normal rats (*p = 0.0198). These outcomes confirmed the detrimental impacts of cisplatin on the hippocampus (Figure 8).

## 4 Discussion

We attempted to clarify the molecular mechanisms underlying the cognitive deficits induced by cisplatin. We focused on the modulation of neuroinflammation and overexpression of glutamate receptors within the hippocampus. Our findings indicate that cisplatin can harm neurons through neuroinflammation and overactivation of AMPARs and NMDARs. These actions result in excessive excitation linked to mitochondrial dysfunction which, ultimately, leads to neuronal apoptosis.

Mortality rate is a critical factor in assessing the risk of drug toxicity. Recent studies indicate a correlation between cisplatin administration and increased mortality in specific patient populations (Zhang et al., 2012; Ranasinghe et al., 2022). Previous research has demonstrated a link between cisplatin dosage and heightened toxicity, thereby increasing mortality risk (Elmorsy et al., 2024; Fung et al., 2018). The study result revealed a gradual increase in mortality following cisplatin treatment, reaching 40% after 10 days in the animals. This finding is consistent with previous observations regarding cisplatin toxicity (Ranasinghe et al., 2022). Consequently, cisplatin-based chemotherapy regimens necessitate rigorous monitoring and careful management to ensure patient safety and treatment efficacy.

Research has indicated that use of cytotoxic agents such as cisplatin can lead to bodyweight loss despite anticancer efficacy, and that cisplatin-associated toxicities (nephrotoxicity, hepatotoxicity, cardiotoxicity) may affect lipid metabolism and muscle mass (Abd Rashid et al., 2021; Patel et al., 2024). The bodyweight of rats was monitored to assess cisplatin-induced toxicity. The cisplatin group exhibited a notable reduction in bodyweight in contrast with that in the control group, consistent with previous findings demonstrating bodyweight loss and anorexia as primary indicators of cisplatin toxicity.

Cisplatin-induced neurotoxicity (a primary cause of chemotherapy-induced learning loss) is differentiated by significant damage to the CNS (Santos et al., 2020; Das et al., 2020). The capacity of cisplatin to penetrate the blood–brain barrier impacts the hippocampus directly, exacerbating the discharge of inflammatory factors and initiating damage to multiple cell types (Patai et al., 2025). This process modifies hippocampal neurochemistry, and increases neurotransmitter release (Wellenberg et al., 2021; Huo et al., 2018). Emerging evidence strongly suggests that inflammatory pathways (notably upregulation of pro-inflammatory factors such as NF-κB, TNF-α, and IL-6) are key contributors to cognitive decline (Mekhora et al., 2024; Alhowail, 2024). We demonstrated significantly increased levels of these factors in the cisplatin rats matched with those in the normal condition. Furthermore, a chronic raise in the level of these pro-inflammatory mediators in the peripheral circulation can lead to breaching of the blood–brain barrier. This action activates astrocytes and microglia, and triggers a central inflammatory response that ultimately leads to cognitive impairment. Furthermore, research has suggested that systemic inflammation can alter mitochondrial function (Alhowail, 2024), resulting in a deficiency in MCI activity.

Increased concentrations of pro-inflammatory substances such as TNF-α and IL-6, liberated by stimulated microglia and astrocytes, lead to increased synaptic glutamate (Smith et al., 2012; Ishijima and Nakajima, 2021). This increase results from glutamate efflux into the extra-synaptic space and the decreased activity of excitatory amino-acid transporters (which normally clear excess glutamate) (Hansen et al., 2017). The resulting increase in synaptic glutamate, secondary to initial inflammatory changes, causes excessive activation of intra-synaptic ionotropic targets, such as AMPARs and NMDARs, potentially contributing to excitotoxicity. Glutamate interacts with AMPARs and NMDARs, and AMPAR activation can displace magnesium from NMDARs (Hansen et al., 2017). This process results in NMDAR overactivation and excessive influx of Ca^2+^, which can lead to neurotoxicity (Hansen et al., 2017; Rozov and Burnashev, 2016). Chronic activation of intra-synaptic AMPARs and NMDARs caused by inflammation results in receptor desensitization and a decrease in intra-synaptic AMPAR density, whereas extra-synaptic NMDAR signaling remains high (Beaurain et al., 2024). This altered ratio of intra-synaptic receptors contributes to neuronal damage. We demonstrated significantly increased mRNA expression of the *GluA1* AMPAR subunit and *NR2A* and *NR2B* NMDAR subunits in the cisplatin-treated group, thereby elucidating a key mechanism of cisplatin-induced hippocampal neurotoxicity.

OS represents a physiological reaction characterized by an increase in ROS originating from mitochondria, which interferes with cellular respiration (Schieber and Chandel, 2014). ROS overproduction activates Kelch-like ECH-linked protein-1 which, in turn, triggers Nrf-2 activation (Ngo and Duennwald, 2022). This transcription factor moves from the cytol to the nucleus, activating the promoter zone responsible for the transcript of antioxidant enzymes such as SOD and GPX (Bellezza et al., 2018). Our study indicated that increased levels of ROS correlate with heightened levels of Nrf-2 and SOD, suggesting OS in rats induced with cisplatin in contrast with rats in the normal animals. These outcomes imply that the combined increase in levels of ROS, Nrf-2, and SOD serves as a protective mechanism against heightened OS and inflammation in the hippocampus. The increase in neuroinflammation and OS appeared to be linked to the increase in Nrf-2 level, whereas SOD served as a protective mechanism against OS.

Synaptic plasticity underpins learning and memory, having a critical role in memory consolidation (Goto, 2022). Increased levels of cytokines and free radicals induce mitochondrial dysfunction, thereby compromising synaptic integrity: early hallmarks of neurodegeneration and cognitive decline (Klemmensen et al., 2024). We revealed reduced MCI activity due to decreased oxidation of NADH, which correlated with increased hippocampal lipid peroxidation. This phenomenon suggests impaired energy production, which affects neuronal function. Moreover, lipid peroxidation (an indicator of cellular damage) contributes to neuronal loss.

The main strength of our study was that we included behavioral evaluations complemented by biochemical analyses (ELISAs, RT-qPCR) aimed at exploring the molecular pathways underlying the cognitive deficits induced by cisplatin. The main limitation was reliance on RT-qPCR for assessing glutamate receptors without evaluating the corresponding protein expression. This constraint arose due to the ELISA kit we acquired failing to react with the protein introduced into the kit.

## 5 Conclusion

Cisplatin mediates cognitive dysfunction, as shown by testing behavioral tasks using the YMT and NORT. Cisplatin use is also associated with hippocampal damage through neuroinflammation (NF-κB, TNF-α, IL-6), increased lipid peroxidation, decreased activity of MCI, and increased levels of mRNA expression in AMPARs and NMDARs. Understanding this hippocampal damage is critical for mounting therapeutic interferences for people suffering from malignancy.

## Data Availability

The original contributions presented in the study are included in the article/supplementary material, further inquiries can be directed to the corresponding author/s.
